# Takotsubo Cardiomyopathy Mimicking Multivessel Coronary Artery Disease Following Spinal Surgery

**DOI:** 10.7759/cureus.61795

**Published:** 2024-06-06

**Authors:** Dolores Sanchez Morey, Samer Kholoki

**Affiliations:** 1 Medicine, Saint James School of Medicine, The Valley, AIA; 2 Internal Medicine, La Grange Memorial Hospital, Chicago, USA

**Keywords:** multivessel disease, multivessel coronary artery disease, diagnostic challenges, stress-induced myocardial infarction, spinal surgery, takotsubo cardiomyopathy

## Abstract

Takotsubo cardiomyopathy (TC), also known as "broken-heart syndrome," is a reversible form of left ventricular dysfunction predominantly affecting post-menopausal women. This case report describes a 62-year-old female who presented with dyspnea and chest pain six weeks after left transforaminal lumbar decompression and fusion surgery. Despite clinical findings suggestive of multivessel coronary artery disease (MVD), angiography revealed normal coronary arteries, confirming TC and stress-induced myocardial infarction. The patient's clinical course highlights the importance of recognizing TC's diverse clinical presentations, especially following surgical interventions, and underscores the need for individualized diagnostic approaches and treatment strategies. The case emphasizes the role of ongoing monitoring and research to understand TC's pathophysiology and optimize therapeutic management.

## Introduction

Multivessel coronary artery disease (MVD) of the heart, characterized by blockages or narrowing in multiple coronary arteries, poses significant cardiovascular risks and complications [1]. Screening programs targeting patients with coronary artery disease (CAD) have been developed to predict the risk of developing multivascular diseases, with factors like age, diabetes, and CAD severity playing significant roles [2].

Studies indicate that MVD is linked to increased mortality rates in patients with ST-elevation myocardial infarction (STEMI) undergoing primary percutaneous coronary intervention (PCI) [3]. The prevalence of MVD in CAD patients varies between 28% and 40%, with factors like age, diabetes, and smoking predicting its occurrence [4]. In stable ischemic heart disease patients, MVD is prevalent and linked to poorer outcomes, including higher mortality and a greater risk of recurrent myocardial infarction compared to those with single-vessel disease [5].

Takotsubo cardiomyopathy (TC), also known as transient apical ballooning syndrome or broken heart syndrome, is a type of non-ischemic cardiomyopathy that primarily affects post-menopausal women. It is characterized by temporary weakening of the left ventricle without significant blockage in the coronary arteries or acute plaque rupture. Unlike typical heart conditions, abnormal wall motion in TC often affects areas beyond a single coronary artery's territory [6].

The pathophysiology of TC involves various mechanisms. Elevated catecholamines from stress can trigger microvascular spasms and direct heart muscle injury [7]. Additionally, factors like microvascular dysfunction, inflammation, estrogen deficiency, coronary vessel spasms, and myocardial infarction are implicated in TC [8]. Estrogen, known for its cardioprotective effects, may contribute to TC in post-menopausal women by inducing exaggerated vasoconstriction and altered stress responses [9]. The complex interplay of these factors underscores the multifactorial nature of TC, highlighting the need for further research to elucidate the exact pathogenesis of this condition and develop targeted therapeutic strategies.

The exact prevalence of TC is unclear due to potential misdiagnosis and its similarity to acute coronary syndrome (ACS) without advanced diagnostic tools. In the United States, based on hospital discharge records, the prevalence of TC was 5.2 per 100,000 for females and 0.6 per 100,000 for males, accounting for 0.02% of hospitalizations. The annual incidence of TC hospitalizations rose from 5.7 per 100,000 person-years in 2007 to 17.4 per 100,000 in 2012. Patients with TC often had CAD, with TC identified in 2.1% of female ACS patients and 0.5% of all ACS patients in a 2014 study [10].

The report aims to highlight the challenges in diagnosing and managing TC in patients with multiple comorbidities and recent surgical interventions. Additionally, it emphasizes the importance of comprehensive clinical evaluation, appropriate diagnostic testing, and tailored therapeutic strategies in the management of complex cardiovascular conditions. This case serves to expand the understanding of the diverse clinical presentations of TC and underscores the significance of considering this diagnosis in patients with atypical cardiac symptoms, especially in the context of recent surgical procedures and underlying medical conditions.

## Case presentation

A 62-year-old female presented for a follow-up appointment at her primary care physician's office to re-evaluate her pain management after undergoing a left transforaminal lumbar decompression and fusion at levels L3-L5. During the encounter, the patient reported the new onset of dyspnea on exertion and chest pain localized to the right mid-clavicular area radiating to the mid-axillary area. The patient stated she started noticing this new onset in the past week, and it was specifically exacerbated when going up the stairs to her apartment. The patient's medical history was significant for type 2 diabetes managed with metformin, empagliflozin, and semaglutide, hypothyroidism post complete thyroidectomy managed with levothyroxine, and hyperlipidemia managed with Lipitor.

**Figure 1:**
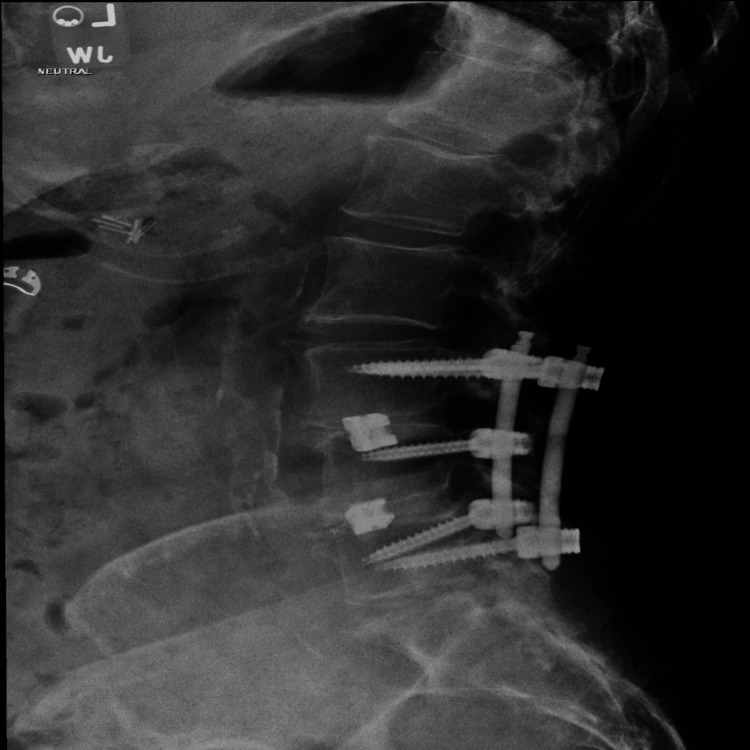
An X-ray of the lumbar spine (lateral view); left transforaminal lumbar fusion L3-L5 is visualized.

**Figure 2:**
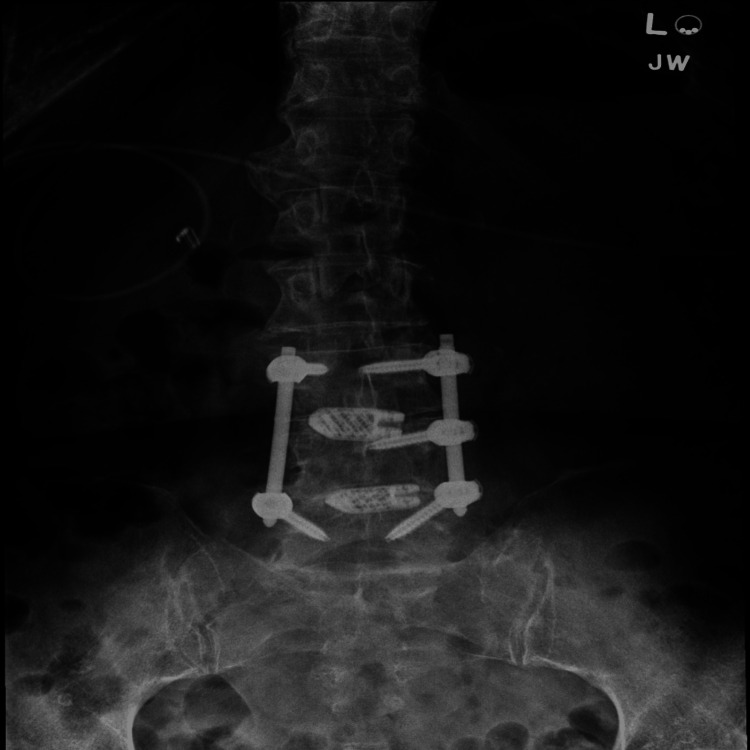
An X-ray of the lumbar spine (anteroposterior view); left transforaminal lumbar fusion L3-L5 is visualized

Six weeks prior to presentation at her primary care physician's office, the patient underwent a left transforaminal lumbar decompression and fusion for worsening spondylolisthesis, disk degeneration, lumbar spondylosis, and stenosis. The surgical procedure involved inferior L3 and L4 facetectomy, full laminectomy at L3-4 and L4-5, diskectomies at L3-4 and L4-5, arthrodesis of the endplates, placement of titanium 3D-printed cages from NuVasive (NuVasive, Inc., San Diego, CA) at L3-4 and L4-5, and non-segmental pedicle screw fixation bilaterally at L3 to L5, skipping the right L4 pedicle (Figures 1, 2). 

**Figure 3:**
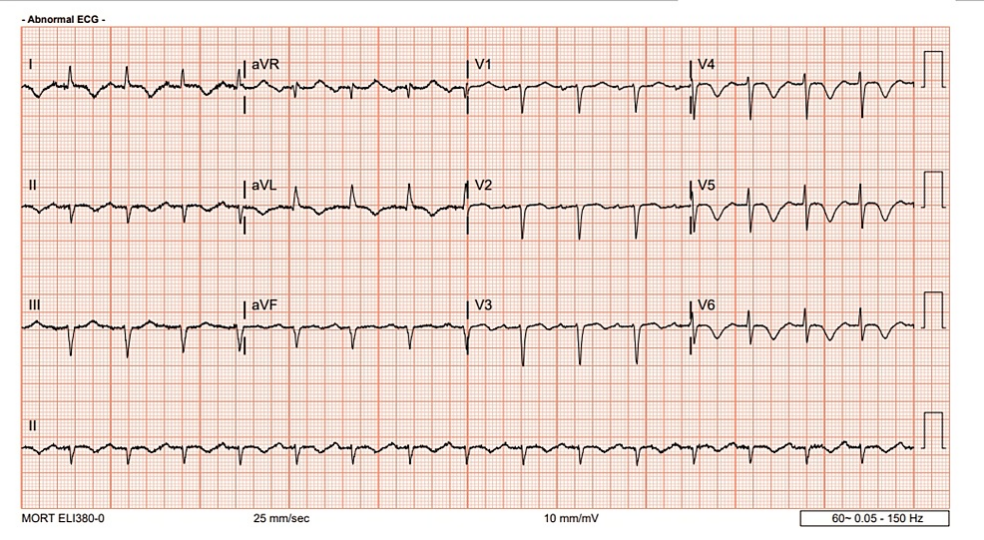
An EKG revealed diffuse ST elevations and T-wave inversions with left-axis deviation

On examination, the patient's vital signs were stable, and a physical examination revealed 2+ bilateral lower extremity pitting edema, mild jugular venous distension (JVD), and a mild S3 on auscultation. An electrocardiogram (EKG) performed showed diffuse ST elevations and T-wave inversions with a left-axis deviation (Figure 3). Due to the findings, the patient was subsequently transferred to the ED. Subsequent EKGs in the emergency department confirmed these findings. Laboratory investigations revealed a normal troponin level, an elevated D-dimer level, and an elevated brain natriuretic peptide 32 (BNP).

Given the elevated D-dimer and strong suspicion of pulmonary emboli, a chest computed tomography (CT) scan with contrast was performed, revealing two small pulmonary emboli in the right lung. The patient was started on intravenous heparin as per protocol. The patient was later admitted to the hospital, and a transthoracic echocardiogram (TTE) was performed before a cardiac angiogram to view the left ventricle function. The TTE revealed left ventricular systolic dysfunction with an ejection fraction of 30%-35% and hypokinesis of the mid-apical myocardium. These findings were suggestive of either MVD or takotsubo (stress) cardiomyopathy. The right ventricular systolic function was normal, and a small pericardial effusion was identified adjacent to the right side of the heart. The intraventricular septum was poorly seen but slightly dilated with possible mild diastolic right ventricular/right atrial compression.

Subsequently, the patient underwent a cardiac angiogram due to the strong suspicion of MVD due to her comorbidities. Cardiac angiograms revealed angiographically normal coronary arteries and a normal left ventricular end-diastolic pressure of around 12, confirming the alternate diagnosis of TC and a stress-induced myocardial infarction. The patient was started on a new cardiac regimen comprising an angiotensin-converting enzyme (ACE) inhibitor, a beta-blocker, and an alternating double diuretic.

**Figure 4:**
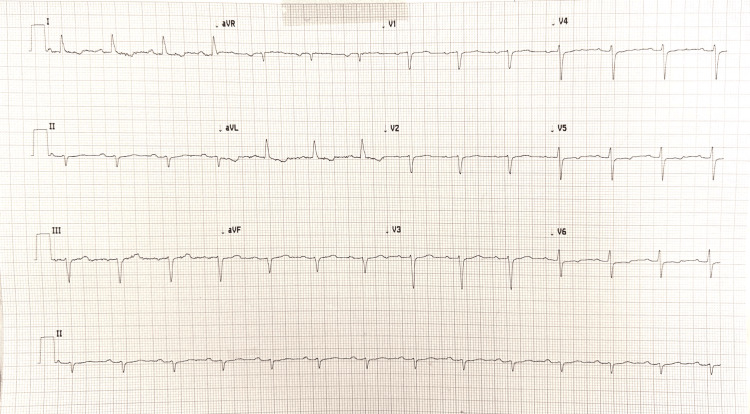
An EKG revealed no significant ST elevations, no T-wave inversions, and a mild left-axis deviation.

Three weeks after discharge, the patient presented to her primary care physician's office to re-evaluate her management for TC. The patient reported significant improvement in her chest pain and admitted that her dyspnea on exertion was now mild. On her physical examination, no bilateral lower extremity pitting edema was noted, no JVD was noted, and her mild S3 previously heard on auscultation was absent. Another EKG was performed, which revealed no significant ST elevations, improved T-wave inversions, and mild left-axis deviation (Figure 4). The patient is set to have another TTE in the near future to continue monitoring her left ventricle dysfunction. The patient was advised to continue her current cardiac regimen of ACE inhibitors, beta-blockers, and alternating double diuretics. The patient was provided with adequate education regarding the prognosis of TC, and a reasonable time frame of 10-12 months was set for this patient to return to baseline parameters. 

## Discussion

Multivessel coronary artery disease of the heart can be associated with TC, a condition characterized by reversible left ventricular dysfunction without obstructive CAS. While TC is commonly triggered by emotional or physical stress, it can also involve epicardial coronary vasospasm [11]. The distinction between TC and ACS can be challenging, especially when there is coexisting CAD. Pathophysiological mechanisms of TC include transient vascular injury followed by myocardial inflammatory changes, often triggered by aberrant post-β2-adrenoceptor signaling and catecholamine release [8,12]. Experimental models suggest that regional differences in vascular gene expression, particularly in the endothelin pathway, may underlie the basis of TC, highlighting the complex nature of this syndrome [13].

The literature on myocardial ischemia as a cause of TC lacks extensive data on epicardial coronary artery vasospasm. Few cases of confirmed vasospasm have been reported, mostly involving single coronary arteries rather than diffuse multivessel vasospasm. Studies using provocative testing have supported the vasospastic theory, with some showing positive vasospasm in TC patients [11,14].

In a previously reported case, a 54-year-old woman presented with chest pain and was diagnosed with TC attributed to diffuse multivessel coronary artery vasospasm. This vasospasm was likely induced by psychosocial stress following the sudden and tragic death of her grandchild. The case highlighted the role of vasospastic disease in TC and underscored the importance of targeted pharmacological treatment, including calcium-channel blockers, beta-blockers, and long-acting nitrates, in managing such cases [11].

Takotsubo cardiomyopathy can be triggered by emotional or physical stress, including surgeries, and can manifest as reversible left ventricular dysfunction resembling ACS [15,16]. Stressors such as surgeries can induce TC by causing myocardial injury through the release of endothelial neurotransmitters during stress [7,8].

Takotsubo cardiomyopathy's perioperative occurrence and varied clinical presentations have been observed in other case studies. An 80-year-old patient from Peru developed TC with cardiogenic shock post-surgery for Chilaiditi’s syndrome [15]. Another case reported TC induced by psychological and physical stress after a successful PCI. [16] Additionally, TC was observed post surgical excision of an adrenal gland tumor [17] and after liver transplant (LT) in patients with alcoholic cirrhosis and high doses of epinephrine during LT [18]. These cases, alongside our presented case, underscore the importance of recognizing TC's diverse clinical manifestations and predisposing factors, emphasizing the need for vigilant diagnosis and tailored management strategies, especially in the perioperative setting.

Takotsubo cardiomyopathy can occur following spinal surgery, as evidenced by cases reported in various contexts. For instance, a 67-year-old woman developed TC during a lumbar discectomy [19]. Additionally, TC has been linked to acute hydrocephalus after posterior fossa tumor resection, causing cardiac dysfunction in a two-year-old girl [20]. These cases highlight the importance of considering TC as a potential complication following spinal surgeries, necessitating awareness among healthcare providers for timely diagnosis and management. Our case, in contrast, presents a delayed onset of TC, emphasizing the need to consider this diagnosis even weeks after surgical interventions.

## Conclusions

Our case highlights TC's unique presentation as a multivessel, non-obstructive disease following spinal surgery, emphasizing the importance of considering this diagnosis even weeks post-procedure. This underscores the need for ongoing monitoring and tailored management strategies for TC, especially in patients with underlying comorbidities. Given the infrequent occurrence of coronary vasospasm in TC, treatment often relies on individual symptom assessment and addressing potential triggers. Continued research is essential to better understand TC's mechanisms and optimize therapeutic approaches.
