# Correction: Takotsubo Cardiomyopathy Mimicking Multivessel Coronary Artery Disease Following Spinal Surgery

**DOI:** 10.7759/cureus.c181

**Published:** 2024-06-07

**Authors:** Dolores Sanchez Morey, Samer Kholoki

**Affiliations:** 1 Medicine, Saint James School of Medicine, The Valley, AIA; 2 Internal Medicine, La Grange Memorial Hospital, Chicago, USA

This article has been corrected at the request of the submitting author to change her affiliation as follows:

Dolores Sanchez Morey Original: Medicine, Saint James School of Medicine, Chicago, USA Corrected: Medicine, Saint James School of Medicine, Anguilla, AIA

Samer Kholoki Original: Internal Medicine, La Grange Memorial Hospital, Chicago, USA Internal Medicine, Saint James School of Medicine, Chicago, USA Corrected: Internal Medicine, La Grange Memorial Hospital, Chicago, USA

The authors deeply regret that these errors were not identified and addressed prior to publication.

 

